# Phonetic changes, dental occlusion and their relationships in individuals with cleft lip and palate undergoing orthognathic surgery

**DOI:** 10.1590/2317-1782/e20230139en

**Published:** 2025-12-12

**Authors:** Melissa Picinato-Pirola, Andressa Sharllene Carneiro da Silva, Bruna Mara Adorno Marmontel Araújo, Ana Paula Fukushiro

**Affiliations:** 1 Faculdade de Ciências e Tecnologias em Saúde, Universidade de Brasília – UnB - Brasília (DF), Brasil.; 2 Hospital de Reabilitação de Anomalias Craniofaciais – HRAC, Universidade de São Paulo – USP - Bauru (SP), Brasil.; 3 Departamento de Fonoaudiologia, Faculdade de Odontologia de Bauru – FOB, Universidade de São Paulo – USP - Bauru (SP), Brasil.

**Keywords:** Cleft Palate, Orthognathic Surgery, Malocclusion, Malocclusion, Angle Class III, Speech Disorders, Rhinomanometry, Nasometry

## Abstract

**Purpose:**

To verify changes in phonetic, nasometric and aerodynamic aspects of speech in individuals with cleft lip and palate and the influence of occlusion on these variables, before and after orthognathic surgery (OC) for maxillary advancement.

**Methods:**

Retrospective, observational, cross-sectional and analytical study. Fifty-one patients with previously repaired cleft lip and palate participated, 26 men and 25 women (x̅=31 years), who underwent maxillary advancement OC. The following were performed: assessment of phonetic and occlusal aspects, nasometry and aerodynamic assessment of velopharyngeal function (flow-pressure technique), before and after OC. The phonetic aspects were evaluated by 3 experienced judges.

**Results:**

There was high intra and inter judge agreement. There was a significant improvement in phonetic production after OC: in tongue interposition ([d], [t], [l], [n], [ʎ]), distortion ([f], [v]) and anterior lisp ([s], [z], [ʃ]). In the occlusal aspects, the overjet reached, on average, the normal values after the OC and there was an occlusal improvement in the anterior crossbite, openbite and overbite. Nasalance values were within the normal range after CO for the nasal text and there was an increase in nasalance, suggestive of hypernasality, in the oral text. There was no change in velopharyngeal closure, in the production of the word “rampa”, suggestive of adequate velopharyngeal closure. Occlusion did not influence nasalance and velopharyngeal closure before or after maxillary advancement OC.

**Conclusion:**

There was a significant improvement in phonetic, occlusal and nasometric aspects after OC. However, none of the phones obtained 100% adequacy,reinforcing the importance of speech therapy after OC.

## INTRODUCTION

Occlusal changes may be present in cases of cleft lip and palate, especially when the maxilla and alveolar process are affected (pre-incisive foramen or trans-incisive foramen clefts). Many patients tend to exhibit a Class III occlusal pattern, with anterior and/or posterior crossbite, due to maxillary growth deficiency caused by primary surgeries. This condition can be corrected in adulthood through orthognathic surgery (OS)^(1-4)^.

Regarding speech alterations, in addition to compensatory articulations and hypernasality—which are common among patients with cleft lip and palate—distortions^(5,6)^ or changes in the place of articulation may also be observed. These are often related to occlusal changes, particularly affecting labiodental, linguodental, and alveolar sounds^(7)^. In a study involving 8-year-old children with repaired cleft lip and palate and altered inter-arch dental relationship^(8)^, 69% of the children exhibited lisping during speech production. The study concluded that dentofacial deformities may contribute to the occurrence of lisping in the cleft lip and palate population.

In another study^(7)^, which evaluated the speech of individuals with cleft lip and palate before and after orthognathic surgery (OS) in relation to place of articulation, it was observed that prior to surgery, patients exhibited severe speech impairment—especially in the production of labiodental, linguodental, and alveolar phones (characterized by bilabial placement, interdental articulation, and lisping). After OS, there was an improvement in speech regarding the degree of impairment. Studies show that the maxillomandibular adjustment provided by OS favors the positioning of the teeth and tongue, and consequently, speech production^(6,9-11)^.

Patients undergoing OS may experience other effects beyond the phonetic alterations already mentioned. Maxillary advancement surgery (Le Fort I) can increase the velopharyngeal space between the soft palate and the posterior pharyngeal wall. This increase may be compensated by the lateral pharyngeal walls and palatal musculature^(12,13)^. Thus, although OS has a potentially beneficial effect on speech due to the restoration of maxillomandibular balance, it may also lead to the onset or worsening of pre-existing hypernasality in patients with cleft palate^(14-17)^.

One of the main methods for evaluating speech is auditory-perceptual assessment^(18,19)^. However, it is a subjective method that depends on the evaluators’ experience and carries the risk of intra- and/or inter-evaluator disagreement^(20,21)^. Although clinical assessment of speech resonance performed by speech-language pathologists is considered the “gold standard,” the oronasal balance alterations associated with cleft lip and palate and velopharyngeal dysfunction are difficult to evaluate, and there may be low agreement among listeners regarding their perceptual-auditory evaluations.^19^ Therefore, the use of instrumental methods is important to complement clinical assessment, allowing for better planning and monitoring of therapeutic procedure outcomes^(18,20,22,23)^.

Nasometry is one of the instrumental methods used to estimate speech nasality through the measurement of nasalance. It is non-invasive, easy to operate, and calculates the percentage ratio between acoustic data collected by two microphones—one nasal and one oral. This percentage is referred to as nasalance. Thus, the nasometer quantifies the relative acoustic energy of speech and complements perceptual impressions of nasality with objective data, assisting clinicians in making inferences about the adequacy of velopharyngeal function in speech, as well as in obtaining information related to upper airway obstruction^(24)^.

Another instrument for objectively evaluating velopharyngeal function is aerodynamic speech assessment using the pressure-flow technique^(25)^. This is a direct and precise examination that allows for the evaluation of velopharyngeal closure during speech by simultaneously measuring nasal airflow and oral and nasal air pressures within the vocal tract. This assessment is based on the principle that the cross-sectional area of an opening can be estimated by measuring the pressure on both sides of the constriction along with the airflow passing through it^(25)^. Studies have demonstrated the effectiveness of this technique in assessing the velopharyngeal mechanism^(26,27)^.

According to existing literature, there is a lack of studies examining the occlusal influence on phonetic, nasometric, and aerodynamic aspects of speech in Brazilian Portuguese-speaking individuals with cleft lip and palate before and after (OS). It is considered that diagnosing phonetic, nasometric, and aerodynamic speech alterations - alongside aesthetic aspects - is essential for determining the need for secondary surgery and/or speech therapy. Therefore, this study aims to establish classifications for phonetic, nasality, and aerodynamic speech alterations, contributing to the profiling of patients in the pre- and postoperative phases of OS. Additionally, the influence of occlusion on these aspects was evaluated before and after surgery, given the expected occlusal improvement following the procedure. By contributing to a more accurate diagnosis, the study supports treatment planning to prevent and minimize alterations at each stage.

The objective of the present study was to examine the changes in phonetic, nasometric, and aerodynamic aspects of speech in individuals with cleft lip and palate, as well as the influence of occlusion on these aspects, before and after surgical maxillary advancement.

## METHODS

This was a retrospective, observational, cross-sectional, and analytical study, approved by the institution’s Human Research Ethics Committee (CAAE: 54031221.3.0000.5441, approval no.: 5.262.190). All participants signed an Informed Consent Form.

**Figure 1:**
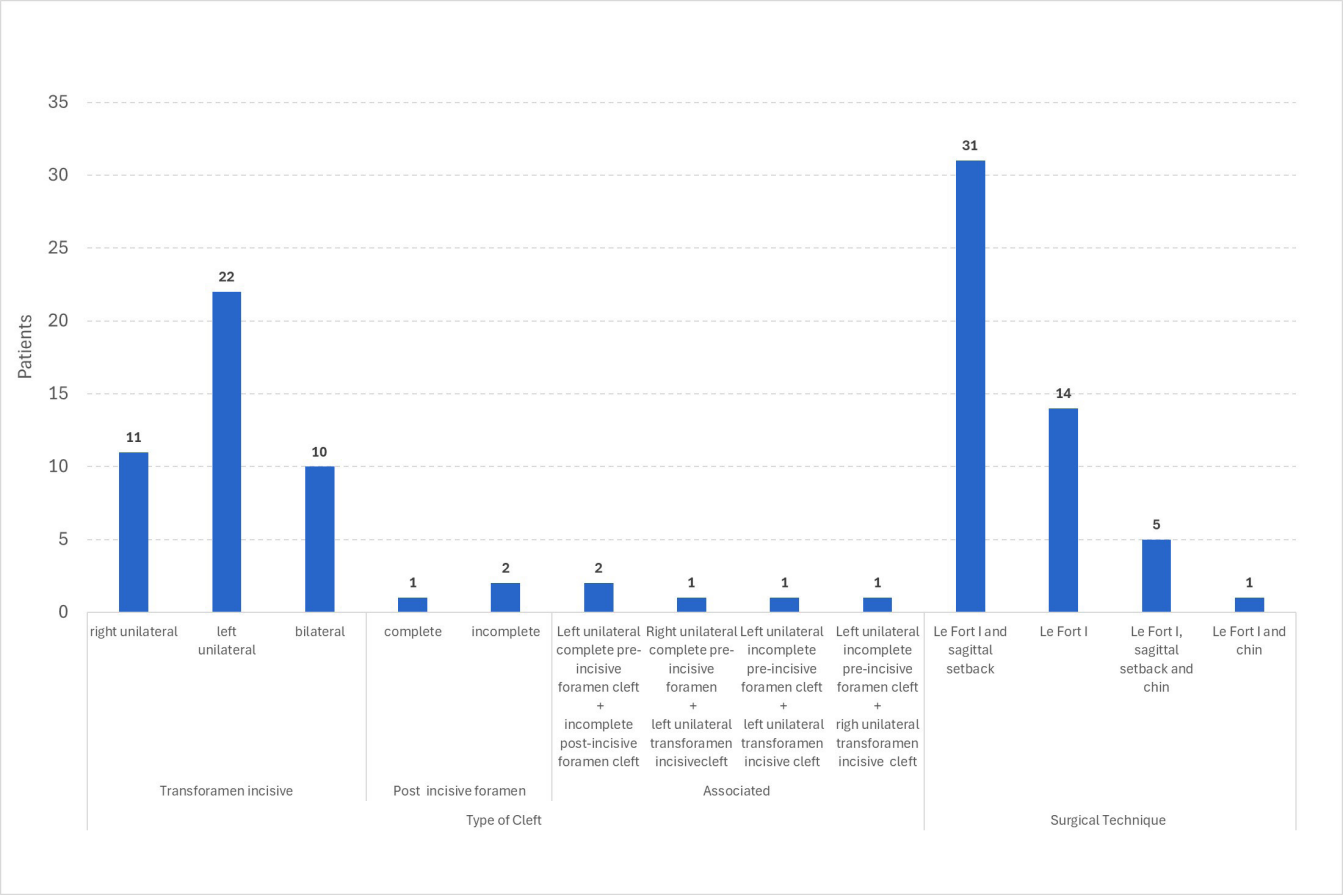
Distribution of study participants by type of cleft lip and palate and orthognathic surgical technique

The study sample was selected from a pre-existing database at the Laboratory of Physiology of the Hospital for Rehabilitation of Craniofacial Anomalies at the University of São Paulo (HRAC-USP). Initially, 168 medical records were analyzed. From these, 51 records of patients with repaired cleft lip and palate were selected according to inclusion and exclusion criteria—26 male and 25 female patients, with a mean age of 31 (±5.2) years. All had undergone maxillary advancement orthognathic surgery (Le Fort I), with or without involvement of the mandibular segment. The classifications of cleft lip and palate types and orthognathic surgery techniques are presented in Figure 1.

Inclusion criteria included patients aged 18 years or older with speech, resonance, nasometric, and aerodynamic evaluations with good audiovisual recording quality. Patients were excluded if their medical records indicated any type or degree of neurological impairment, presence of syndromes, fistulas in the hard and/or soft palate region, history of facial trauma, or prior head and neck surgeries (except for cheiloplasty and/or palatoplasty).

Data were obtained during clinical and instrumental evaluations, following the routine protocol of the Laboratory of Physiology at HRAC - USP. This included assessments of phonetic and occlusal aspects, as well as nasometric and aerodynamic speech evaluations. Records were selected from both the pre- and postoperative phases of OS.

Examinations were typically conducted about one day before OS, between 60 to 90 days after the procedure for speech and occlusal assessments, and approximately 12 months post-surgery for instrumental evaluations of nasometry and aerodynamic aspects of speech. As HRAC-USP is a specialized center for cleft lip and palate treatment, patients originated from various and often distant regions, and were therefore referred for speech therapy in their hometowns—a variable that could not be controlled.

### Assessment of phonetic and occlusal aspects

Speech and occlusal samples were obtained through clinical evaluation using the Orofacial Myofunctional Assessment Protocol for Individuals with Cleft Lip and Palate (PROTIFI)^(28)^. General clinical history data and video recordings of patients reading specific phrases for each phone^(7)^ (Chart 1) were used. For audiovisual recording, a JVC digital camcorder, model GY-HM150E, was used, connected to a Sony microphone, model ECM-MS957, mounted on a stand positioned 40 cm from the patient’s mouth. The camera was placed on a tripod at a distance of 1 meter from the patient. Recordings were conducted in a soundproof room.

**Chart 1:** Speech sample used for phonetic analysis^(7)^

1) A perna do pássaro é pequena. [ p ]
2) Ela é a babá do bebê. [ b ]
3) O tatu estava na toca. [ t ]
4) Odete é a dona da padaria. [ d ]
5) Lucas comeu queijo. [ k ]
6) Guilherme é banguela e legal. [ g ]
7) O primeiro tamanco era de camurça. [ m ]
8) O número do ônibus é noventa e nove. [ n ]
9) Há minhocas no ninho da Aninha. [ ɲ ]
10) Afonso ofendeu o chefe. [ f ]
11) A avó do Vitor é malvada e nervosa. [ v ]
12) Cássia passeou com Sônia. [ s ]
13) O casaco da Zezé é azul. [ z ]
14) Chico deixou o caixote na chuva. [ ʃ ]
15) Rogério tem jóias e relógios. [ Ʒ ]
16) O relógio de Lívia é colorido. [ l ]
17) As mulheres colheram o melhor milho. [ ʎ ]
18) No porão há baratas e pererecas. [ ɾ ]
19) O corredor da rua de barro é reto. [ R ]

Pre- and postoperative speech samples were edited using specialized software, coded, distributed, and independently evaluated by three speech-language pathologists, each with at least 10 years of experience in diagnosing and treating speech disorders resulting from cleft lip and palate. A total of 122 samples were distributed for analysis: 51 preoperative, 51 postoperative, 10 repeated preoperative samples, and 10 repeated postoperative samples, to assess intra-rater agreement. The samples were randomly divided into two evaluation phases, with 61 samples in phase 1 and 61 in phase 2, and a 20-day deadline for analysis in each phase, which was respected by all judges.

For speech aspect classification, the judges recorded either absence (score 0) or presence (score 1) of the following: compensatory articulations, obligatory disorders, distortion, anterior lisp, lateral lisp, and tongue interposition, as outlined in Chart 2.

**Chart 2:** Instructions for the classification of phonetic alterations

Phonetic Alteration	Description	Classification
Compensatory articulations	Compensatory productions involving constrictive movements that may occur in the larynx, pharynx, velum, palate, and/or nasopharyngeal cavity.	Assign a score of (0) in the absence and a score of (1) in the presence of any compensatory articulation.
Obligatory disorders	When nasal snoring, weak intraoral pressure, nasal air emission, and/or hypernasality are present.	Assign a score of (0) in the absence and a score of (1) in the presence of obligatory disorders.
Distortion	There is a change in the production of the phoneme’s articulatory point, meaning a modification in its execution.	Assign a score of (0) in the absence and a score of (1) in the presence of distortion.
Anterior lisping	Occurs in the pronunciation of the phonemes [s], [z], [ʃ], and [Ʒ], with the tongue advanced/interposed between the central and lateral incisors.	Assign a score of (0) in the absence and a score of (1) in the presence of anterior lisping.
Lateral lisping	Incorrect tongue positioning occurs during the production of the phonemes [s], [z], [ʃ], and [Ʒ]. The tongue remains retracted with possible air escape. It may also occur with the tongue interposed between the posterior teeth.	Assign a score of (0) in the absence and a score of (1) in the presence of lateral lisping.
Tongue interposition	The tongue remains interposed between the anterior teeth (central and lateral incisors), generally occurring in the phonemes [t], [d], [l], and [n]. However, it may occur in other phonemes except for fricatives, which are classified as anterior lisping.	Assign a score of (0) in the absence and a score of (1) in the presence of tongue interposition.

Occlusal aspects were analyzed based on intraoral examination data from the PROFITI protocol^(28)^, including vertical overlap (overbite) and horizontal overlap (overjet). Normality criteria included: proper midline alignment (0 mm), vertical and horizontal overlap up to 3 mm, absence of transverse alterations, and absence of fixed prostheses.

### Nasometric evaluation

Nasometric evaluation was conducted during the preoperative period and approximately one year after OS, using either the Nasometer model 6200-2 or the Nasometer II model 6400. The values recorded in the patient's nasometric assessment protocol were identified for this study. Both devices consist of a system in which the oral and nasal acoustic energies of speech are captured by two microphones—one directed at the mouth and the other at the nose—separated by a horizontal metal plate positioned above the upper lip during the recording of standardized speech samples. The signal from each microphone is filtered and digitized by electronic modules and processed by specialized software that calculates nasalance. The nasalance values obtained correspond to a numerical ratio between the nasal sound pressure level (SPL) and the total SPL (sum of oral and nasal SPL), multiplied by 100. The speech stimuli used for nasometric evaluation included the reading of two sets of phrases: one oral text (exclusively oral sounds) and one nasal text (predominantly nasal sounds), as proposed by Trindade et al.^(22)^.

The nasalance values collected from the two speech stimuli were analyzed descriptively using cutoff values proposed in the literature to interpret and group findings into indicators of hypernasality (nasalance >27% for oral text) and hyponasality (nasalance <43% for nasal text)^(22)^.

After classifying speech nasality in both evaluation modalities, these cutoff values were used to assess the effect of resonance conditions (balanced, hypernasal, hyponasal) for both speech stimuli (oral and predominantly nasal), in both the preoperative and postoperative periods.

### Aerodynamic speech evaluation

This evaluation was based on the principle of a technique that estimates the smallest cross-sectional area of an opening by simultaneously measuring the pressure on both sides of the constriction and the airflow passing through it^(25)^. Using the pressure-flow technique, velopharyngeal function can be estimated by measuring the minimum cross-sectional area of the velopharyngeal opening during speech, through the computerized system PERCI-SARS 3.50 (Microtronics Corp).

The velopharyngeal area was determined during the production of the word “rampa,” by positioning one catheter inside the oral cavity and another, held by an obturator, in the nostril with lower nasal airflow, as determined by Glatzel’s mirror analysis. Both the oral cavity catheter and the nasal catheter measured air pressures, which were transmitted through pressure transducers. Nasal airflow was measured using a plastic tube adapted to the nostril with greater airflow, connected to a heated pneumotachograph and linked to a flow transducer.

Signals from the three transducers—oral pressure, nasal pressure, and nasal airflow—were sent to the PERCI system for analysis using specialized software. The area calculation was obtained using the equation A = V / K × √(2∆P / d), where A is the area of the opening in cm^2^, V is the nasal airflow in ml/s, K is 0.65, ∆P is the difference between oral and nasal pressure in cmH_2_O, and d is the air density in g/cm^3^.

Velopharyngeal closure was classified according to the proposed values^(25)^: a velopharyngeal area from 0 to 0.049 cm^2^ was considered adequate; from 0.050 to 0.099 cm^2^, borderline-adequate; from 0.100 to 0.199 cm^2^, borderline-inadequate; and equal to or greater than 0.200 cm^2^, inadequate.

### Statistical analysis

Statistical analysis was performed using SPSS software, version 23. All differences were considered statistically significant at a 5% significance level.

Descriptive statistical analyses were calculated for all variables and expressed as frequency and percentage for categorical variables, and as mean, minimum, and maximum for numerical variables. To assess inter- and intra-rater agreement in the evaluation of phonetic aspects before and after surgery, the Intraclass Correlation Coefficient (ICC) was used. For the phonetic evaluation, 51 patients were considered, each with 19 speech sample phrases recorded at two time points (pre- and post-OS), resulting in a total of 1,938 phrases evaluated by three judges, yielding a total sample of 5,814 phrases. To assess phonetic improvement in isolated phonemes after surgery, the median score from the three judges was used, representing the central value of their evaluations. To analyze improvements in phonetic and occlusal aspects after surgery by comparing pre- and postoperative periods, the McNemar test was applied for categorical variables and the paired Student’s t-test for numerical variables with means. To verify the influence of vertical and horizontal occlusal changes on phonetic alterations, the chi-square test was used.

To analyze nasalance and aerodynamic speech evaluation across pre- and postoperative periods, the McNemar test was again applied. To assess the influence of occlusion on nasometric evaluation, the Mann-Whitney test was used, and for aerodynamic speech evaluation, the Spearman correlation test was applied.

## RESULTS

### Phonetic and occlusal aspects

In the verification of inter- and intra-rater agreement in the evaluation of phonetic aspects before and after surgery, the inter-rater ICC was found to be 89.9% among the three judges, with an ICC of 94.1% between judges 1 and 2, 92.1% between judges 1 and 3, and 93.5% between judges 2 and 3. In the intra-rater evaluations, the ICC for judge 1 was 95.4%, for judge 2 was 97.6%, and for judge 3 was 97.9%.

**Figure 2:**
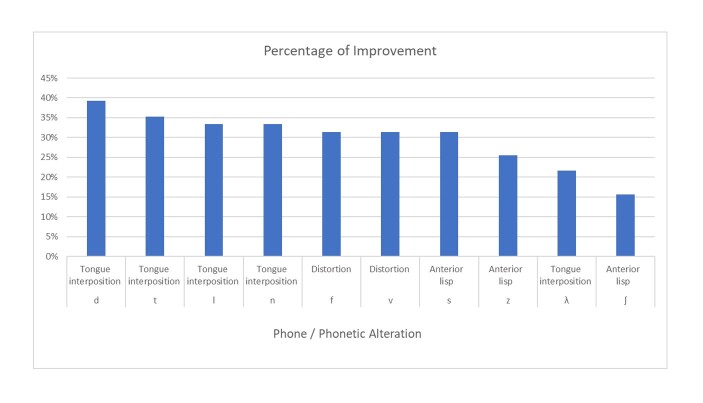
Highest improvement percentages found in phonetic alterations by phone in the pre- and post-orthognathic surgery comparison

A significant improvement was observed in phonetic aspects when comparing patients before and after surgery: in tongue interposition ([d]=39%, [t]=35%, [l]=33%, [n]=33%, /ʎ/=22%), distortion ([f]=31%, [v]=31%), and anterior lisping ([s]=31%, [z]=25%, [ʃ]=16%). Obligatory disorders did not show a significant difference when analyzed individually by sound; however, a significant difference was found when comparing the total number of patients, as a greater number of individuals presented symptoms after surgery. No significant improvement was observed in compensatory articulations and lateral lisping (Table 1 and Figure 2).

**Table 1:** Comparison of the presence of phonetic alterations in 51 patients before and after orthognathic surgery evaluated by three judges

Phone	Before – Compensatory Articulation	After – Compensatory Articulation	Before – Obligatory Errors	After – Obligatory Errors	Before – Distortion	After – Distortion	Before – Anterior Lisping	After – Anterior Lisping	Before – Lateral Lisping	After – Lateral Lisping	Before – Tongue Interposition	After – Tongue Interposition
p	0	0	4	9	0	0	0	0	0	0	0	0
b	0	0	4	9	0	0	0	0	0	0	0	0
t	0	0	3	8	2	1	0	0	0	0	25	8*
d	0	0	3	8	1	1	0	0	0	0	27	8*
k	0	1	3	5	0	0	0	0	0	0	0	0
g	0	0	3	6	0	0	0	0	0	0	0	0
m	0	0	0	0	0	0	0	0	0	0	0	0
n	0	0	0	0	0	0	0	0	0	0	25	8^*^
ɲ	0	0	0	0	0	0	0	0	0	0	0	0
f	0	0	4	4	22	9*	0	0	0	0	0	0
v	0	0	4	3	21	7*	0	0	0	0	0	0
s	1	1	5	4	0	0	23	8*	7	7	0	0
z	0	0	4	4	0	1	20	7*	4	4	0	0
ʃ	0	0	4	4	0	0	9	2*	8	3	0	0
Ʒ	0	0	5	5	0	0	8	2	8	3	0	0
l	0	0	2	2	0	1	0	0	0	0	29	13*
ʎ	0	0	2	1	0	0	0	0	0	0	13	4*
ɾ	0	0	2	1	2	2	0	0	0	0	1	0
R	0	0	3	2	0	0	0	0	0	0	0	0
Total	1	2	55	75*	48	22*	60	19*	27	17	120	41*

Statistical test: McNemar

* p≤0.05

Regarding occlusal aspects, a statistically significant difference was observed in the comparison of horizontal overlap before and after OS. Additionally, there was a statistical difference in occlusal aspects when comparing pre- and postoperative conditions related to both vertical and horizontal overlap, except in edge-to-edge bite (Table 2). For this analysis, 48 patients were considered, as 3 did not have records in their medical charts.

**Table 2:** Pre- and post-orthognathic surgery analysis of vertical overlap, horizontal overlap, and dento-occlusal alterations

	Vertical overlap (mm)			N without occlusal alterations		N with edge-to-edge bite		N with increased vertical overlap (deep bite)		N with open bite (N)		N Total
	Minimum	Maximum	Mean									
pre	-7.60	6.44	1.37	28	58%	2	4%	10	21%	8	17%	48
post	-1.33	3.56	1.89	43*	90%	1	2%	2*	4%	2*	4%	48
	Horizontal overlap (mm)			N without occlusal alterations		N with edge-to-edge bite		N with increased horizontal overlap (overjet)		N with anterior crossbite (N)		N Total
	Minimum	Maximum	Mean									
pre	-14.80	13.15	-4.54	3	6%	0	0%	3	6%	42	88%	48
post	-1.99	5.55	2.67*	27^*^	56%	1	2%	19*	40%	1*	2%	48

Paired Student's t-test (analysis of the mean vertical and horizontal overlap before and after surgery)

McNemar statistical test (analysis of occlusion before and after surgery)

* p≤0.05

Caption: mm: millimeters; N: number of patients

Vertical occlusal changes did not influence the majority of phone productions that showed significant improvement in phonetic aspects when comparing patients before and after OS. A significant difference was observed only in tongue interposition for the phone [λ] and in distortion of the phone [f] (Table 3).

**Table 3:** Vertical occlusal changes in individuals who presented phonetic alterations and their influence on phoneme production

Phonetic Alterations			N without occlusal alterations	N with edge-to-edge bite	N with increased vertical overlap (deep bite)	N with open bite	P value
Tongue interposition	[t]	pre	13	0	4	7	0.10
		post	6	0	0	1	0.46
	[d]	pre	14	0	5	7	0.07
		post	7	0	0	1	0.53
	[l]	pre	16	0	4	7	0.07
		post	11	0	0	2	0.08
	[n]	pre	13	0	5	6	0.24
		post	7	0	0	1	0.53
	[ʎ]	pre	6	0	4	2	0.56
		post	2	0	0	2	<0.01^*^
Distortion	[f]	pre	11	1	5	3	0.92
		post	6	0	0	2	0.01*
	[v]	pre	11	1	4	3	0.99
		post	5	0	0	1	0.38
Anterior lisping	[s]	pre	13	0	3	5	0.31
		post	6	0	0	1	0.46
	[z]	pre	10	0	2	6	0.06
		post	5	0	0	1	0.38
	[ʃ]	pre	2	0	2	3	0.34
		post	1	0	0	1	0.07

Chi-square statistical test

* p≤0.05

Caption: N: number of patients

Regarding the horizontal occlusal changes, these were found to influence most of the sounds that showed significant improvement in phonetic aspects after OS (Table 4).

**Table 4:** Horizontal occlusal changes in individuals who presented phonetic alterations and their influence on sound production

Phonetic Alterations			N without occlusal alterations	N with edge-to-edge bite	N with increased horizontal overlap (overjet)	N with anterior crossbite	P value
Tongue interposition	[t]	pre	0	0	1	23	#
		post	2	0	4	1	0.05*
	[d]	pre	0	0	1	25	#
		post	4	0	3	1	0.15
	[l]	pre	0	0	0	27	#
		post	6	1	5	1	0.12
	[n]	pre	0	0	0	24	#
		post	4	0	3	1	0.15
	[ʎ]	pre	0	0	0	12	#
		post	1	1	1	1	<0.01*
Distortion	[f]	pre	0	0	1	19	#
		post	2	1	4	1	0.00*
	[v]	pre	0	0	1	18	#
		post	2	1	3	0	0.04*
Anterior lisping	[s]	pre	0	0	2	19	#
		post	3	0	3	1	0.09
	[z]	pre	0	0	0	18	^#^
		post	2	0	3	1	0.04^*^
	[ʃ]	pre	0	0	0	7	#
		post	1	0	0	1	0.001*

Chi-square statistical test

* p≤0.05;

# statistical analysis was not possible

Caption: N: number of patients

### Nasometry

In the nasometric evaluation of speech, a statistically significant difference (p≤0.05) was observed in both nasal and oral texts when comparing the pre- and postoperative periods (Table 5).

**Table 5:** Evaluation of nasalance and speech aerodynamics in the pre- and postoperative periods

Nasalance	Nasal		P value	Oral		P value
	pre	post		pre	post	
Balanced oronasal resonance	29 (56.9%)	42 (82.4%)	*<0.001	45 (88.2%)	33 (64.7%)	^*^0.003
Hyponasality	22 (43.1%)	9 (17.6%)		-	-	
Hypernasality	-	-		6 (11.8%)	18 (35.3%)	
Aerodynamic evaluation	/gradient/ pre-surgical			/gradient/ post-surgical		P value
Adequate VPF	37 (73%)			32 (63%)		0.174
Borderline adequate VPF	7 (14%)			9 (18%)		
Borderline inadequate VPF	5 (10%)			6 (12%)		
Inadequate VPF	2 (4%)			4 (8%)		

McNemar statistical test

* p≤0.05

Caption: VPF: velopharyngeal closure

### Speech aerodynamics

The aerodynamic assessment of speech in both pre- and postoperative periods showed no statistically significant difference (p≤0.05), as presented in Table 5.

### Influence of occlusal aspects on nasometric and aerodynamic speech evaluations

No influence was observed between occlusion (vertical and horizontal overlap) and nasometric evaluation (p>0.05, Mann-Whitney test).

In the application of Spearman’s correlation test, no correlation was found between occlusal aspects and the aerodynamic evaluation of speech during the production of the word “rampa.” The results of Spearman’s correlation between aerodynamic evaluations and preoperative vertical overlap (r=-0.30), postoperative vertical overlap (r=0.12), preoperative horizontal overlap (r=-0.10), and postoperative horizontal overlap (r=0.05) all showed p>0.05.

## DISCUSSION

Occlusal alterations are common in patients with cleft lip and palate involving the maxilla and the alveolar process, contributing to the development of a Class III dentofacial deformity, which is generally corrected through OS^(1-4)^. This dentofacial deformity leads to articulatory adjustments in speech to compensate for morphological changes, resulting in speech impairments such as sound distortions and lisps^(5-8)^. The retrusion and growth deficiency of the upper arch in relation to the mandibular arch in Class III dentofacial deformity can affect tongue positioning during the production of alveolar sounds due to tongue protrusion, crossbite, maxillary constriction, and misaligned teeth^(5)^.

In this study, tongue interposition, distortions, anterior and lateral lisping were observed, along with obligatory disorders and compensatory articulations in the evaluated patients with cleft lip and palate. The most relevant findings that showed significant improvement in phonetic production after OS were tongue interposition and anterior lisping. Existing literature indicates that fricative sounds are the most affected in individuals with dentofacial deformities^(8,29)^, which aligns with this study, as all fricative phones presented anterior lisping, including the alveolar [s] and [z], and the alveolopalatal [ʃ] and [Ʒ]; with the phone [Ʒ] showing no significant improvement after surgery. Among the fricatives, distortion was also observed in the labiodental sounds [f] and [v], as noted in another study^(7)^. Improvement was also observed after surgery in tongue interposition for the alveolar plosive [t] and [d], the alveolar nasal [n], the alveolar lateral [l], and the palatal lateral [ʎ]. It is believed that the occlusal changes following surgery contributed to the improvement in sound production.

It is known that patients with dentofacial deformities present occlusal and muscular alterations that impair the performance of orofacial functions^(5,6)^. Patients with Class III dentofacial deformity show a higher prevalence of articulatory errors and consonant distortions when compared to individuals without such alterations, and the severity of malocclusion may correlate with the degree of speech distortion^(11)^. One of the objectives of this study was to determine whether the phonetic alterations presented by patients with cleft lip and palate before OS improved after the procedure. According to this analysis, a significant improvement was observed, indicating that the occlusal correction provided by OS had a positive impact on the speech of patients who exhibited tongue interposition, distortions, and anterior lisping. However, despite the statistically significant improvement, the maximum percentage of improvement was 39% for tongue interposition in the phone [d], meaning that no sound improved 100% solely through the occlusal adjustment provided by OS, highlighting the importance of speech therapy after surgery.

The study by Alaluusua et al.^(6)^ evaluated the effect of maxillary advancement on the Finnish alveolar consonants [s], [l], and [ɾ] in 59 patients with cleft lip and palate who underwent OS. There was significant improvement in the phone [s] (34% of patients showed alteration in preoperative production and 20% in postoperative), and in [l] (34% of patients showed alteration preoperatively and 19% postoperatively), while [ɾ] showed no difference after maxillary advancement. The authors concluded that when planning OS in patients with cleft lip and palate with maxillary retrusion and phonetic alterations, maxillary advancement may be a means to improve articulation of the phones [s] and [l], and they emphasized the need for speech therapy following surgery.

Another phonetic alteration investigated was the obligatory errors, which is a consequence of velopharyngeal dysfunction and is characterized by the presence of hypernasality, weak intraoral pressure, and nasal air emission^(25)^. In the statistical analysis of the present study, no significant difference was observed when obligatory errors were compared individually by sound; however, a significant difference was noted when comparing the total number of patients, meaning that after OS, a greater number of patients presented this condition. Studies show that following maxillary advancement surgery, patients may experience the onset or worsening of hypernasality^(14-17)^.

It is also noted that a smaller number of patients exhibited lateral lisping after surgery, although this difference was not statistically significant. It is estimated that different results may be found in future studies conducted with a larger sample size.

The patients who participated in the study showed a low presence of compensatory articulations, a fact that may be explained by the inclusion of adult patients who had already undergone primary surgeries for the correction of cleft lip and palate, as well as other interventions that may have helped overcome or prevented the development of compensatory articulations. Another hypothesis is that these patients already had adequate velopharyngeal closure before OS, which may have contributed to the absence of compensatory articulations. It is known that approximately 30% of children with cleft lip and palate present velopharyngeal dysfunction, which can lead to hypernasality, weak intraoral pressure, nasal air emission, and compensatory articulations^(30)^.

Regarding occlusal aspects, no significant difference was found when comparing the mean vertical overlap before and after surgery, indicating that most patients had average vertical overlap values within normal limits both before and after surgery (mean before: 1.37; mean after: 1.89). However, it is important to highlight that patients who presented occlusal alterations related to vertical overlap measurements, such as open bite (8 patients, 17%) and deep bite (10 patients, 21%), showed significant improvement in occlusion after surgery, with only 2 patients (4%) continuing to present open bite and deep bite postoperatively.

A similar outcome was observed in the comparison of horizontal overlap before and after surgery, where anterior crossbite occlusal alterations (42 patients, 88%) were corrected after surgery, showing significant improvement, with only 1 patient (2%) presenting anterior crossbite postoperatively. Regarding increased horizontal overlap (overjet), initially 3 patients presented this alteration, and after surgery, 19 patients (40%) showed it. This result is related to the surgical correction of Class III dentofacial deformity. The repositioning of the maxillary and/or mandibular segment may have caused an increase in horizontal overlap with measurements above 3 mm, which are considered altered. Furthermore, patients improved their average horizontal overlap compared to the preoperative average (mean before surgery: -4.54 and after: 2.67 – within normal values). Patients in this study who presented edge-to-edge bite did not show significant occlusal changes when comparing pre- and postoperative periods.

In a systematic review of patients without cleft lip and palate who underwent OS, it was found that increased horizontal overlap (overjet) decreased in patients with skeletal Class III dentofacial deformity after surgery and in long-term follow-up. Open bite (overbite) increased in patients with Class II dentofacial deformity, while Class III patients showed variable results^(31)^.

In this study, it was also observed that vertical occlusal changes influenced the sounds [ʎ] and [f] after surgical correction. Regarding horizontal occlusal changes, the occlusal adjustment provided by surgery improved the production of the phones [t], [ʎ], [f], [v], [z], and [ʃ]. Although the literature does not contain studies that focus exclusively on these aspects, it is known that discrepancies in vertical and horizontal overlap can negatively affect speech through distortions^(11)^. It is hypothesized that, despite the sample size, significant differences were found mainly related to the surgical correction of horizontal overlap, which deserves further exploration in future studies with a larger number of individuals to determine whether more phonemes show significant differences in these aspects.

In the study, objective nasometry and aerodynamic speech evaluations were used. In the comparison of nasometric speech evaluation before and after OS, a statistically significant difference (p≤0.05) was observed in both the predominantly nasal and oral texts, as previously reported in other studies^(3,14)^, which also showed an increase in hypernasality in the oral text after maxillary advancement. For the aerodynamic speech evaluation, the word “rampa” was used during the pre- and postoperative periods, and no statistically significant difference (p≤0.05) was found. In the study by Alaluusua et al.^(3)^, the risk of patients with cleft palate developing velopharyngeal inadequacy after maxillary advancement was higher in those who already showed symptoms of velopharyngeal inadequacy before undergoing OS. In that study, 73% of patients had adequate velopharyngeal closure before surgery, and 63% after, resulting in no statistically significant difference.

Researchers conducted a study^(14)^ with the aim of evaluating the impact of OS on nasalance and the aerodynamic assessment of speech in patients with cleft lip and palate. The repaired patients were evaluated at three time points: before surgery, 45 days after, and 9 months after. A significant increase in average nasalance was observed at both 45 days and 9 months postoperatively, compared to the preoperative measurements of oral sentences and nasal phrases. In the aerodynamic speech evaluation, a significant increase in the minimum velopharyngeal closure area was observed after 45 days, and a significant increase in the average cross-sectional area was noted after 9 months of OS, with 73% of patients no longer presenting subnormal areas seen in the preoperative phase. It was concluded that, in the long term, OS modifies the nasalance of some patients with cleft, which may be justified by the increase in nasal size, potentially improving nasal airflow for breathing.

In another study^(19)^, an analysis was conducted on the nasalance scores of individuals with cleft palate who underwent maxillary advancement surgery. The authors concluded that the onset of hypernasality after OS in individuals with cleft palate, whether or not involving the lip, occurred in similar proportions regardless of the type of cleft.

In a recent study by the same group of researchers^(13)^, a survey was conducted over the past 20 years involving 535 patients from a reference center for the treatment of cleft lip and palate. The objective was to assess the effect of OS on nasal dimensions and correlate them with respiratory symptoms. It was observed that after OS, there was a significant increase in the nasal cross-sectional area evaluated by anterior and posterior rhinomanometry, although no significant increase was found in the nasopharyngeal cross-sectional area. Regarding respiratory symptoms after OS, 26.3% of patients showed improvement in nasal obstruction, 28.5% in oronasal breathing, 18.5% in snoring, and 5.2% in respiratory obstruction during sleep.

In a systematic review^(23)^, the authors aimed to evaluate speech and changes in velopharyngeal function in patients with cleft palate after maxillary advancement surgery. It was found that the effect of maxillary advancement on speech and velopharyngeal closure remains controversial and warrants further structured studies.

In this research, no influence was observed between occlusion (vertical and horizontal overlap) and nasometric evaluation, nor between occlusal aspects and the aerodynamic evaluation of speech during the production of the word “rampa.” It is believed that the occlusal changes provided by OS did not influence objective nasometry and aerodynamic speech evaluations, confirming the initial hypothesis that occlusion affects phonetic aspects (lisps and distortions), which depend on tooth positioning, but not nasality and velopharyngeal closure, which depend on muscular structures located in the nasopharyngeal region.

This is a pioneering study that explored changes in phonetic, nasometric, and aerodynamic aspects of speech in individuals with cleft lip and palate, as well as the influence of occlusion on these aspects before and after maxillary advancement surgery. It presents a comprehensive analysis of the production of 19 sounds to examine the alterations and their modifications resulting from maxillary advancement. In addition, objective nasometry and aerodynamic speech evaluations were analyzed in patients who underwent maxillary advancement, correlating these findings with occlusal aspects, which was a distinctive feature of this study.

Thus, the profile of the patient before and after maxillary advancement surgery is established, which will support treatment planning, minimizing and preventing alterations at each stage. These findings are believed to fill a gap in the literature, although future studies with a larger sample of patients for each type of cleft lip and palate may be conducted to investigate possible statistical differences not identified in this study.

## CONCLUSION

There were changes in phonetic aspects following OS. The phonetic alterations that showed significant improvement after OS included tongue interposition in the phones [d], [t], [l], [n], [ʎ]; distortion in the phones [f] and [v]; and anterior lisping in the phones [s], [z], and [ʃ]. Regarding occlusal aspects, horizontal overlap reached normal values after OS, and there was occlusal improvement in anterior crossbite, open bite, and deep bite.

In the comparison of nasometric evaluation, balanced resonance predominated after OS for the nasal text, and there was an increase in hypernasality in the oral text following surgery. There was no change in velopharyngeal closure during the production of the word “rampa,” which remained adequate both before and after surgery.

Occlusion did not influence nasalance or velopharyngeal closure either before or after maxillary advancement surgery.

It is important to emphasize that although there was improvement in phonetic production following occlusal adjustment through surgical correction, no phone achieved 100% adequacy, reinforcing the importance of speech therapy after CO.
