# Supplementary material for: Phonetic changes, dental occlusion and their relationships in individuals with cleft lip and palate undergoing orthognathic surgery
Source: Codas. 2025 Dec 12;37(6):e20230139. doi: 10.1590/2317-1782/e20230139en (PMC12704614; doi:10.1590/2317-1782/e20230139en)
Supplement: Translation

# Translation: Alterações fonéticas, oclusão dentária e suas relações nos indivíduos com fissura labiopalatina submetidos à cirurgia ortognática

**DOI:** 10.1590/2317-1782/e20230139pt

**Authors:** Melissa Picinato-Pirola, Andressa Sharllene Carneiro da Silva, Bruna Mara Adorno Marmontel Araújo, Ana Paula Fukushiro

**Affiliations:** 1 Faculdade de Ciências e Tecnologias em Saúde, Universidade de Brasília – UnB - Brasília (DF), Brasil.; 2 Hospital de Reabilitação de Anomalias Craniofaciais – HRAC, Universidade de São Paulo – USP - Bauru (SP), Brasil.; 3 Departamento de Fonoaudiologia, Faculdade de Odontologia de Bauru – FOB, Universidade de São Paulo – USP - Bauru (SP), Brasil.

**Keywords:** Fissura Palatina, Cirurgia Ortognática, Má Oclusão, Má Oclusão Classe III De Angle, Distúrbios da Fala, Rinomanometria, Nasometria

## Abstract

**Objetivo:**

Verificar as modificações dos aspectos fonéticos, nasométricos e aerodinâmicos da fala em indivíduos com fissura labiopalatina e a influência da oclusão sobre essas variáveis, antes e após a cirurgia ortognática (CO) de avanço de maxila.

**Método:**

Estudo retrospectivo, observacional, transversal e analítico. Participaram 51 pacientes com fissura labiopalatina previamente reparada, 26 homens e 25 mulheres (x̅=31 anos), submetidos à CO de avanço maxilar. Foram realizados: avaliação dos aspectos fonéticos e oclusais, nasometria e avaliação aerodinâmica da função velofaríngea (técnica fluxo-pressão), antes e após a CO. Os aspectos fonéticos foram avaliados por 3 juízes experientes.

**Resultados:**

Houve alta concordância intra e inter juízes. Observou-se melhora significativa na produção fonética, após a CO: na interposição de língua ([d], [t], [l], [n], [ʎ]), distorção ([f], [v]) e ceceio anterior ([s], [z], [ʃ]). Nos aspectos oclusais, o trespasse horizontal atingiu, em média, os valores de normalidade após a CO e houve melhora oclusal da mordida cruzada anterior, mordida aberta e sobremordida. Os valores de nasalância encontraram-se dentro dos valores de normalidade após CO para o texto nasal e houve aumento da nasalância, sugestiva de hipernasalidade, no texto oral. Não houve modificação no fechamento velofaríngeo, na produção do vocábulo “rampa”, sugestivo de adequado fechamento velofaríngeo. A oclusão não influenciou a nasalância e o fechamento velofaríngeo antes ou após a CO de avanço de maxila.

**Conclusão:**

Houve melhora significativa dos aspectos fonéticos, oclusais e nasométricos após a CO. Entretanto, em nenhum fone se obteve 100% de adequação, reforçando a importância da fonoterapia após a CO.

## INTRODUÇÃO

As alterações oclusais podem estar presentes na fissura labiopalatina, principalmente nos casos que acometem a maxila e o processo alveolar (pré-forame incisivo ou transforame incisivo). Muitos pacientes tendem a apresentar um padrão oclusal Classe III, com mordida cruzada anterior e/ou posterior, devido à deficiência do crescimento maxilar, ocasionado pelas cirurgias primárias, a qual pode ser corrigida em idade adulta, por meio de cirurgia ortognática (CO)^(1-4)^.

No que se refere às alterações de fala, além das articulações compensatórias e hipernasalidade, que muitos pacientes com fissura labiopalatina apresentam, também podem ser observadas distorções^(5,6)^ ou alterações de ponto articulatório, devido às alterações oclusais, principalmente nos fones labiodentais, linguodentais e alveolares^(7)^. Em um estudo com crianças de 8 anos com fissura labiopalatina operada e alteração na relação inter-arcos dentários^(8)^, verificou-se presença de ceceio na produção da fala em 69% das crianças, concluindo que as deformidades dentofaciais podem favorecer a ocorrência de ceceio na população com fissura labiopalatina.

Em outro estudo^(7)^, que avaliou a fala de indivíduos com fissura labiopalatina antes e após a CO em relação ao ponto articulatório, observou-se que os pacientes, antes da cirurgia apresentavam comprometimento grave da fala, principalmente nos fones labiodentais, linguodentais e alveolares (caracterizados por ponto bilabial, interdental e ceceio) e, após a CO, houve melhora na fala em relação ao seu grau de comprometimento. Estudos apontaram que o ajuste maxilomandibular proporcionado pela CO, favorece a posição dos dentes e da língua; e consequentemente, a produção da fala^(6,9-11)^.

Os pacientes que são submetidos à CO, podem apresentar outros acometimentos além das alterações fonéticas já citadas. A CO de avanço de maxila (Le Fort I) pode promover um aumento do espaço velofaríngeo entre o véu palatino e a parede posterior da faringe, o qual pode ser compensado pelas paredes laterais da faringe e a musculatura palatina^(12,13)^. Assim, embora a CO tenha um efeito potencialmente benéfico na fala devido o restabelecimento do equilíbrio maxilomandibular, ela pode provocar o aparecimento ou agravamento da hipernasalidade pré-existente em pacientes com fissura palatina^(14-17)^.

Um dos principais métodos para se avaliar a fala é a avaliação perceptivo-auditiva^(18,19)^. Porém, é um método subjetivo, que depende da experiência dos avaliadores, com risco de discordância intra e/ou interavaliadores^(20,21)^. Apesar da avaliação clínica da ressonância da fala realizada por fonoaudiólogos ser considerada “padrão ouro”, as alterações do equilíbrio oronasal associadas à fissura labiopalatina e à disfunção velofaríngea são difíceis de avaliar e pode haver uma baixa concordância entre os ouvintes em relação às suas avaliações perceptivo-auditivas^(19)^. Dessa forma, a utilização de métodos instrumentais é importante para complementar a avaliação clínica, a fim de permitir um melhor planejamento e acompanhamento de resultados dos procedimentos terapêuticos^(18,20,22,23)^.

A nasometria é um dos métodos instrumentais que permite estimar a nasalidade da fala a partir da medida da nasalância. Não é invasivo, tem fácil manuseio e calcula em porcentagem a relação entre os dados acústicos obtidos por dois microfones, um nasal e um oral, sendo essa porcentagem denominada nasalância. Assim, o nasômetro quantifica a energia acústica relativa da fala e complementa as impressões perceptivas da nasalidade com dados objetivos, auxiliando os clínicos nas inferências em relação a adequação da função velofaríngea da fala, assim como na obtenção de informações relacionadas à obstrução aérea superior^(24)^.

Um outro instrumento para se avaliar objetivamente a função velofaríngea é a avaliação aerodinâmica da fala, por meio da técnica fluxo-pressão^(25)^. Trata-se de um exame direto e preciso que permite avaliar o fechamento velofaríngeo durante a fala por meio da medida simultânea do fluxo aéreo nasal e das pressões aéreas oral e nasal no trato vocal. Esta avaliação se baseia no fato de que a secção transversa de um orifício pode ser estimada pela medida da pressão entre os dois lados da constrição concomitantemente com o fluxo que o atravessa^(25)^. Estudos mostraram a eficácia da técnica para a avaliação do mecanismo velofaríngeo^(26,27)^.

Na literatura, observa-se ausência de estudos que verifiquem a influência oclusal nos aspectos fonéticos, nasométricos e aerodinâmicos da fala em indivíduos falantes do Português Brasileiro com fissura labiopalatina antes e após a CO. Considera-se que o diagnóstico das alterações fonéticas, nasométricas e aerodinâmicas da fala, além dos aspectos estéticos, são importantes para a definição da necessidade de cirurgia secundária e/ou fonoterapia. Dessa forma, espera-se, com esse estudo, que o estabelecimento das classificações das alterações fonéticas, de nasalidade e aerodinâmicas da fala contribuam para o traçado do perfil do paciente no pré e pós-operatório de CO. Em adição, foi verificado a influência da oclusão nesses aspectos antes e após a CO, uma vez que se espera melhora oclusal após a cirurgia. Contribuindo para um diagnóstico mais preciso, o estudo favorece o tratamento a fim de prevenir e minimizar alterações em cada etapa.

O objetivo do presente estudo foi verificar as modificações dos aspectos fonéticos, nasométricos e aerodinâmicos da fala em indivíduos com fissura labiopalatina e a influência da oclusão nesses aspectos, antes e após a CO de avanço de maxila.

## MÉTODOS

Estudo retrospectivo, observacional, transversal e analítico, aprovado pelo comitê de ética e pesquisa em seres humanos da instituição (CAAE: 54031221.3.0000.5441, parecer no.: 5.262.190) e todos os participantes assinaram o Termo de Consentimento Livre e Esclarecido.

A amostra do estudo foi selecionada de um banco de dados previamente existente no Laboratório de Fisiologia do Hospital de Reabilitação de Anomalias Craniofaciais da Universidade de São Paulo (HRAC-USP). Inicialmente foram analisados 168 prontuários, dentre esses, foram selecionados conforme critério de inclusão e exclusão 51 prontuários de pacientes com fissura labiopalatina operada, sendo 26 homens e 25 mulheres, com idade média de 31(±5,2) anos, todos submetidos à CO de avanço maxilar (Le Fort I), podendo ou não envolver o segmento mandibular. As classificações dos tipos de fissura labiopalatina e técnicas de CO estão distribuídas na Figura 1.

**Figura 1:** Distribuição dos participantes do estudo por tipo de fissura labiopalatina e técnica cirúrgica ortognática

Como critério de inclusão, foram selecionados pacientes com idade igual ou superior a 18 anos, que apresentavam as avaliações de fala, ressonância, nasométrica e aerodinâmica da fala completas e com boa qualidade de gravação em sistema audiovisual; e foram excluídos pacientes que tiverem dados no prontuário de algum tipo/ grau de comprometimento neurológico, presença de síndromes, fístulas na região do palato duro e/ou mole, que tinham sofrido traumas de face ou que tivessem histórico de cirurgia de cabeça e pescoço (exceto as cirurgias de queiloplastia e/ou palatoplastia).

Os dados foram obtidos na ocasião da avaliação clínica e instrumental, conforme protocolo de rotina do Laboratório de Fisiologia do HRAC - USP, incluindo: a avaliação dos aspectos fonéticos e oclusais, nasométrica e aerodinâmica da fala, sendo selecionados registros das avaliações tanto no momento pré como pós CO.

Os exames foram realizados em média 1 dia antes da CO, 60 a 90 dias após a cirurgia (para a avaliação da fala e dos aspectos oclusais) e em média 12 meses após a cirurgia (para as avaliações instrumentais de nasometria e aerodinâmica da fala). Por se tratar de um centro especializado no tratamento de fissura labiopalatina, os pacientes eram de localidades distintas e muitas vezes distantes, assim, foram encaminhados para fonoterapia em suas cidades de origem e essa variável não pôde ser controlada.

### Avaliação dos aspectos fonéticos e dos aspectos oclusais

As amostras de fala e oclusais foram obtidas por meio da avaliação clínica pelo Protocolo de Avaliação Miofuncional Orofacial para Indivíduos com Fissura Labiopalatina (PROTIFI)^(28)^. Foram utilizados os dados gerais de histórica clínica e as gravações em vídeo com a leitura de frases específicas para cada fone^(7)^ (Quadro 1). Para a gravação em sistema audiovisual utilizou-se filmadora digital JVC, modelo GY-HM150E, conectada a um microfone marca Sony, modelo ECM-MS957, posicionado em um suporte a uma distância de 40cm da boca do paciente. Para o posicionamento da câmera, utilizou-se um tripé posicionado a 1m de distância do paciente. As gravações foram realizadas em sala silenciosa e com tratamento acústico.

**Quadro 1:** Amostra de fala utilizada para a análise fonética^(7)^

1) A perna do pássaro é pequena. [ p ]
2) Ela é a babá do bebê. [ b ]
3) O tatu estava na toca. [ t ]
4) Odete é a dona da padaria. [ d ]
5) Lucas comeu queijo. [ k ]
6) Guilherme é banguela e legal. [ g ]
7) O primeiro tamanco era de camurça. [ m ]
8) O número do ônibus é noventa e nove. [ n ]
9) Há minhocas no ninho da Aninha. [ ɲ ]
10) Afonso ofendeu o chefe. [ f ]
11) A avó do Vitor é malvada e nervosa. [ v ]
12) Cássia passeou com Sônia. [ s ]
13) O casaco da Zezé é azul. [ z ]
14) Chico deixou o caixote na chuva. [ ʃ ]
15) Rogério tem jóias e relógios. [ Ʒ ]
16) O relógio de Lívia é colorido. [ l ]
17) As mulheres colheram o melhor milho. [ ʎ ]
18) No porão há baratas e pererecas. [ ɾ ]
19) O corredor da rua de barro é reto. [ R ]

As amostras de fala pré e pós-operatórias foram editadas em programa de computador específico, codificadas, distribuídas e avaliadas por três juízes fonoaudiólogos com, no mínimo, 10 anos de experiência no diagnóstico e tratamento das alterações de fala decorrentes de fissura labiopalatina, de forma independente e randomizadas. Foram distribuídas para análise um total de 122 amostras, sendo 51 pré-operatórias, 51 pós-operatórias, 10 amostras repetidas pré-operatórias e 10 repetidas pós-operatórias para verificar a concordância intrajuiz. As amostras foram distribuídas aleatoriamente em 2 fases para a avaliação, sendo 61 na fase 1 e 61 na fase 2, com um prazo de 20 dias para análise em cada fase, o qual foi respeitado por todos os juízes.

Para a classificação dos aspectos da fala, os juízes consideraram a ausência (escore 0) ou a presença (escore 1) de: articulações compensatórias, distúrbios obrigatórios, distorção, ceceio anterior, ceceio lateral e interposição de língua, conforme Quadro 2.

**Quadro 2:** Instruções para a classificação das alterações fonéticas

Alteração Fonética	Descrição	Classificação
Articulações compensatórias	Produções compensatórias que envolvem movimentos constritivos que podem ser realizados na laringe, faringe, no véu, no palato e/ou na cavidade nasofaríngea.	Colocar o escore (0) no caso de ausência e o escore (1) na presença de qualquer uma das articulações compensatórias.
Distúrbios obrigatórios	Quando há presença de ronco nasal, fraca pressão intraoral, emissão de ar nasal e/ou hipernasalidade.	Colocar o escore (0) no caso de ausência e o escore (1) na presença de distúrbios obrigatórios.
Distorção	Há mudança na produção do ponto articulatório do fonema, ou seja, ocorre uma mudança na sua execução.	Colocar o escore (0) no caso de ausência e o escore (1) na presença de distorção.
Ceceio anterior	Ocorre na pronúncia dos fones [s], [z], [ʃ] e [Ʒ], com a língua anteriorizada/interposta entre os dentes incisivos centrais e laterais.	Colocar o escore (0) no caso de ausência e o escore (1) na presença de ceceio anterior.
Ceceio lateral	Ocorre a posição incorreta da língua na produção dos fones [s], [z], [ʃ] e [Ʒ]. A língua permanece posteriorizada e com a possibilidade de ocorrência de escape de ar. Pode ocorrer, ainda, com a língua interposta entre os dentes posteriores.	Colocar o escore (0) no caso de ausência e o escore (1) na presença de ceceio lateral.
Interposição de língua	A língua permanece interposta entre os dentes anteriores (incisivos centrais e laterais), geralmente ocorre nos fones: [t], [d], [l] e [n]. Porém, pode ocorrer nos demais fones, exceto nos fones fricativos, nos quais classificaremos como ceceio anterior.	Colocar o escore (0) no caso de ausência e o escore (1) na presença de interposição de língua.

Para a análise dos aspectos oclusais considerou-se os dados do exame intraoral de dentes e oclusão do protocolo PROFITI^(28)^, sendo eles: trespasse vertical (overbite), trespasse horizontal (overjet). Como critério de normalidade, foram considerados os valores: linha média adequada (0 mm), trespasse vertical e horizontal até 3 mm, ausência de alteração transversal e ausência de prótese dentária.

### Avaliação nasométrica

A avaliação nasométrica, foi realizada no período pré-operatório e aproximadamente 1 ano após a CO, utilizando o Nasômetro, modelo 6200-2 ou Nasômetro II, modelo 6400. Os valores registrados no protocolo de avaliação nasométrica do paciente foram identificados para este estudo. Ambos os equipamentos consistem em um sistema em que as energias acústicas, oral e nasal, da fala são captadas por dois microfones, sendo um direcionado à boca e o outro ao nariz do paciente e separados por uma placa horizontal de metal posicionada acima do lábio superior durante a gravação das amostras de fala. O sinal de cada um dos microfones é filtrado e digitalizado por módulos eletrônicos e processados por um *software* específico, que calcula a nasalância. Os valores de nasalância obtidos correspondem a uma razão numérica entre o nível de pressão sonora (NPS) nasal e o NPS total (soma do NPS oral e nasal), multiplicada por 100. Os estímulos de fala utilizados para avaliação nasométrica incluíram a leitura de dois conjuntos de frases, denominados texto oral (sons exclusivamente orais) e texto nasal (sons predominantemente nasais), propostos por Trindade et al.^(22)^.

Os valores de nasalância coletados para os estímulos de fala incluídos no estudo (2 textos) foram analisados de forma descritiva usando-se os valores de corte propostos na literatura para interpretação e agrupamento dos achados entre medidas indicativas da presença de hipernasalidade (nasalância >27% para texto oral) e de hiponasalidade (nasalância <43% para texto nasal)^(22)^.

Após classificar a nasalidade de fala nas duas modalidades de avaliação utilizou-se os valores de corte, a fim de verificar o efeito das condições de ressonância (equilibrada, hipernasal, hiponasal), dos dois estímulos de fala (texto oral e predominantemente nasal), tanto no período pré-cirúrgico, como no pós-cirúrgico.

### Avaliação aerodinâmica da fala

Para essa avaliação, baseou-se no princípio da técnica que prevê que a menor área de secção transversa de um orifício pode ser estimada concomitantemente pela medida da pressão entre os dois lados da constrição e do fluxo que o atravessa^(25)^. Dessa forma, por meio da técnica fluxo-pressão, a função velofaríngea pode ser estimada pela medida da área de secção transversa mínima do orifício velofaríngeo durante a fala, por meio do sistema computadorizado PERCI-SARS 3.50 (Microtronics Corp).

A área velofaríngea foi determinada durante a produção do vocábulo “rampa”, posicionando-se um cateter mantido no interior da cavidade oral e outro, mantido por um obturador, na narina de menor fluxo nasal, após análise com o espelho de Glatzel. Tanto o cateter da cavidade oral, como o da narina, mediu as pressões aéreas que foram transmitidas pelos transdutores de pressão. O fluxo aéreo nasal foi medido por um tubo plástico adaptado à narina de maior fluxo, conectado a um pneumotacógrafo aquecido e ligado a um transdutor de fluxo.

Os sinais dos três transdutores, pressão oral, pressão nasal e fluxo nasal, foram enviados ao sistema PERCI para a análise por software específico. O cálculo da área foi obtido pela equação: A=V/K (2∆P/d)1/2, sendo A= área do orifício em cm^2^, V= fluxo nasal em ml/s; k=0,65; ∆P=diferença entre pressão oral e pressão nasal em cmH_2_O; d=densidade do ar em g/cm^3^.

O fechamento velofaríngeo foi classificado de acordo com os valores propostos^(25)^: área velofaríngea (cm^2^) de 0 a 0,049: adequado, de 0,050 a 0,099: adequado-marginal, 0,100 a 0,199: marginal-inadequado, ≥0,200: inadequado.

### Análise estatística

A análise estatística foi realizada por meio do software SPSS, versão 23. Todas as diferenças foram consideradas estatisticamente significativas para um nível de significância de 5%.

As análises estatísticas descritivas foram calculadas para todos as variáveis e foram expressas em frequência e porcentagem para as variáveis categóricas e em média, mínimo e máximo para as variáveis numéricas. Para a verificação da concordância inter e intra avaliadores na avaliação dos aspectos fonéticos pré e pós-operatório, utilizou-se o índice de concordância (ICC). Na avaliação fonética, considerou-se os 51 pacientes, com 19 frases de amostras de fala em dois momentos (pré e pós CO), ou seja, uma amostra de 1938 frases, que foi avaliada por 3 juízes, totalizando uma amostra de 5814 frases. Para avaliar a melhora fonética nos fones isolados após a CO utilizou-se a mediana da avaliação realizada pelos 3 juízes, de modo a considerar o valor do escore central encontrado pelos juízes. Para analisar a melhora dos aspectos fonéticos e oclusais após a CO comparando os períodos pré e pós-operatórios, foi utilizado o teste estatístico McNemar para as variáveis categóricas e o teste T de student pareado para as variáveis numéricas com média. Para verificar a influência das alterações oclusais verticais e horizontais nas alterações fonéticas, utilizou-se o teste qui-quadrado.

Para analisar a nasalância e avaliação aerodinâmica da fala nos períodos pré e pós-operatórios, foi utilizado o teste estatístico McNemar. Para verificar a influência da oclusão na avaliação nasométrica, utilizou-se o teste Mann-Whitney e na avaliação aerodinâmica da fala o teste de correlação de Spearman.

## RESULTADOS

### Aspectos fonéticos e dos aspectos oclusais

Na verificação da concordância inter e intra juízes na avaliação dos aspectos fonéticos pré e pós-operatório, foi verificado o ICC inter juízes obtendo-se o resultado de 89,9% entre os 3 juízes, com um ICC de 94,1% entre os juízes 1 e 2; 92,1% entre os juízes 1 e 3; e 93,5% entre os juízes 2 e 3. Nas avaliações intra juízes, foi verificado o ICC do juíz 1 de 95,4%, do juíz 2 de 97,6% e do juíz 3 de 97,9%.

Observou-se melhora significativa nos aspectos fonéticos comparando os pacientes antes e após a CO: na interposição de língua ([d]=39%, [t]=35%, [l]=33%, [n]/=33%, /ʎ/=22%), distorção ([f]=31%, [v]=31%) e ceceio anterior ([s]=31%, [z]=25%, [ʃ]=16%). Os distúrbios obrigatórios não apresentaram diferença significativa quando comparados isoladamente por fones, porém houve diferença significativa na comparação da soma total de pacientes, uma vez que após a CO um maior número de indivíduos passou a apresentar os sintomas. Não houve melhora significativa quanto às articulações compensatórias e ceceio lateral (Tabela 1 e Figura 2).

**Tabela 1:** Comparação da presença das alterações fonéticas nos 51 pacientes antes e após a cirurgia ortognática avaliados pelos 3 juízes

Fones	Pré_ Articulação compensatória	Pós_ Articulação compensatória	Pré_Distúrbios Obrigatórios	Pós_Distúrbios Obrigatórios	Pré_Distorção	Pós_Distorção	Pré_Ceceio anterior	Pós_Ceceio anterior	Pré_Ceceio lateral	Pós_Ceceio lateral	Pré_Interposição de língua	Pós_Interposição de língua
p	0	0	4	9	0	0	0	0	0	0	0	0
b	0	0	4	9	0	0	0	0	0	0	0	0
t	0	0	3	8	2	1	0	0	0	0	25	8^*^
d	0	0	3	8	1	1	0	0	0	0	27	8*
k	0	1	3	5	0	0	0	0	0	0	0	0
g	0	0	3	6	0	0	0	0	0	0	0	0
m	0	0	0	0	0	0	0	0	0	0	0	0
n	0	0	0	0	0	0	0	0	0	0	25	8*
ɲ	0	0	0	0	0	0	0	0	0	0	0	0
f	0	0	4	4	22	9*	0	0	0	0	0	0
v	0	0	4	3	21	7*	0	0	0	0	0	0
s	1	1	5	4	0	0	23	8*	7	7	0	0
z	0	0	4	4	0	1	20	7*	4	4	0	0
ʃ	0	0	4	4	0	0	9	2*	8	3	0	0
Ʒ	0	0	5	5	0	0	8	2	8	3	0	0
l	0	0	2	2	0	1	0	0	0	0	29	13*
ʎ	0	0	2	1	0	0	0	0	0	0	13	4*
ɾ	0	0	2	1	2	2	0	0	0	0	1	0
R	0	0	3	2	0	0	0	0	0	0	0	0
Total	1	2	55	75*	48	22*	60	19*	27	17	120	41*

Teste estatístico McNemar

* p≤0,05

**Figura 2:** Maiores percentuais de melhora encontrados nas alterações fonéticas por fone na comparação pré e pós CO

Em relação aos aspectos oclusais, observou-se que houve diferença estatística na comparação do trespasse horizontal antes e após a CO. Além disso, houve diferença estatística nos aspectos oclusais comparados antes e após a CO relacionados tanto ao trespasse vertical como horizontal, exceto na mordida em topo (Tabela 2). Para essa análise foram considerados 48 pacientes, pois 3 não apresentavam registro em prontuário.

**Tabela 2:** Análise pré e pós cirurgia ortognática do trespasse vertical, trespasse horizontal e alterações dento-oclusais

	Trespasse vertical (mm)			N sem alterações oclusais		N com mordida topo		N com trespasse vertical aumentado (sobremordida)		N com mordida aberta (N)		N Total
	Mínimo	Máximo	Média									
pré	-7,60	6,44	1,37	28	58%	2	4%	10	21%	8	17%	48
pós	-1,33	3,56	1,89	43*	90%	1	2%	2*	4%	2^*^	4%	48
	Trespasse horizontal (mm)			N sem alterações oclusais		N com mordida topo		N com trespasse horizontal aumentado (*sobressalência)*		N com mordida cruzada anterior (N)		N Total
	Mínimo	Máximo	Média									
pré	-14,80	13,15	-4,54	3	6%	0	0%	3	6%	42	88%	48
pós	-1,99	5,55	2,67*	27*	56%	1	2%	19*	40%	1*	2%	48

Teste estatístico t de student pareado (análise da média do trespasse vertical e horizontal pré e pós cirurgia)

Teste estatístico McNemar (análise da oclusão pré e pós cirurgia)

* p≤0,05

Legenda: mm: milímetros; N: número de pacientes

As alterações oclusais verticais não influenciaram a maioria da produção dos fones que obtiveram melhora significativa nos aspectos fonéticos comparando os pacientes antes e após a CO. Apenas observou-se diferença significativa na interposição de língua do fone [λ] e na distorção do fone [f] (Tabela 3).

**Tabela 3:** Alterações oclusais verticais nos indivíduos que apresentaram alterações fonéticas e sua influência na produção do fonema

Alterações Fonéticas			N sem alterações oclusais	N com mordida topo	N com trespasse vertical aumentado (sobremordida)	N com Mordida aberta	P valor
Interposição de língua	[t]	pré	13	0	4	7	0,10
		pós	6	0	0	1	0,46
	[d]	pré	14	0	5	7	0,07
		pós	7	0	0	1	0,53
	[l]	pré	16	0	4	7	0,07
		pós	11	0	0	2	0,08
	[n]	pré	13	0	5	6	0,24
		pós	7	0	0	1	0,53
	[ʎ]	pré	6	0	4	2	0,56
		pós	2	0	0	2	<0,01^*^
Distorção	[f]	pré	11	1	5	3	0,92
		pós	6	0	0	2	0,01*
	[v]	pré	11	1	4	3	0,99
		pós	5	0	0	1	0,38
Ceceio anterior	[s]	pré	13	0	3	5	0,31
		pós	6	0	0	1	0,46
	[z]	pré	10	0	2	6	0,06
		pós	5	0	0	1	0,38
	[ʃ]	pré	2	0	2	3	0,34
		pós	1	0	0	1	0,07

Teste estatístico qui-quadrado

* p≤0,05

Legenda: N: número de pacientes

Em relação às alterações oclusais horizontais, observou-se que estas influenciaram a maioria dos fones que tiveram melhora significativa nos aspectos fonéticos após a CO (Tabela 4).

**Tabela 4:** Alterações oclusais horizontais nos indivíduos que apresentaram alterações fonéticas e sua influência na produção do fonema

Alterações Fonéticas			N sem alterações oclusais	N com mordida topo	N com trespasse horizontal aumentado (*overjet*)	N com mordida cruzada anterior	P valor
Interposição de língua	[t]	pré	0	0	1	23	#
		pós	2	0	4	1	0,05*
	[d]	pré	0	0	1	25	#
		pós	4	0	3	1	0,15
	[l]	pré	0	0	0	27	#
		pós	6	1	5	1	0,12
	[n]	pré	0	0	0	24	#
		pós	4	0	3	1	0,15
	[ʎ]	pré	0	0	0	12	#
		pós	1	1	1	1	<0,01*
Distorção	[f]	pré	0	0	1	19	#
		pós	2	1	4	1	0,00*
	[v]	pré	0	0	1	18	#
		pós	2	1	3	0	0,04*
Ceceio anterior	[s]	pré	0	0	2	19	#
		pós	3	0	3	1	0,09
	[z]	pré	0	0	0	18	#
		pós	2	0	3	1	0,04*
	[ʃ]	pré	0	0	0	7	^#^
		pós	1	0	0	1	0,001^*^

Teste estatístico qui-quadrado

* p≤0,05;

# não foi possível analisar estatisticamente

Legenda: N: número de pacientes

### Nasometria

Na avaliação nasométrica da fala, observou-se diferença estatisticamente significativa (p≤0,05) tanto no texto com predominância nasal, como oral, na comparação dos períodos pré e pós-operatório (Tabela 5).

**Tabela 5:** Avaliação da nasalância e aerodinâmica da fala nos períodos pré e pós-operatórios

Nasalância	Nasal			P valor	Oral		P valor
	Pré	Pós			Pré	Pós	
ressonância equilibrada	29 (56,9%)	42 (82,4%)		*<0,001	45 (88,2%)	33 (64,7%)	^*^0,003
hiponasalidade	22 (43,1%)	9 (17,6%)			-	-	
hipernasalidade	-	-			6 (11,8%)	18 (35,3%)	
Avaliação aerodinâmica	/rampa/ pré- cirúrgico				/rampa/ pós-cirúrgico		P valor
FVF adequado	37 (73%)				32 (63%)		0,174
FVF adequado marginal	7 (14%)				9 (18%)		
FVF marginal inadequado	5 (10%)				6 (12%)		
FVF inadequado	2 (4%)				4 (8%)		

Teste estatístico McNemar

* p≤0,05

Legenda: FVF: fechamento velofaríngeo

### Aerodinâmica da fala

Na avaliação aerodinâmica da fala na produção do vocábulo “rampa” nos períodos pré e pós-operatórios, não houve diferença estatisticamente significativa (p≤0,05) conforme apresentado na Tabela 5.

### Influência dos aspectos oclusais nas avaliações nasométrica e aerodinâmica da fala

Não se observou influência entre a oclusão (trespasse vertical e horizontal) e avaliação nasométrica (p>0,05, teste Mann-Whitney).

Na aplicação do teste de correlação de Spearman, não houve correlação dos aspectos oclusais e a avaliação aerodinâmica da fala na produção do vocábulo “rampa”. Foi observado como resultado da correlação de Spearman entre as avaliações aerodinâmicas e o trespasse vertical pré (r=-0,30), trespasse vertical pós (r=0,12), trespasse horizontal pré (r=-0,10) e trespasse horizontal pós (r=0,05); p>0,05.

## DISCUSSÃO

As alterações oclusais são comuns em pacientes com fissura labiopalatina com o acometimento da maxila e do processo alveolar, favorecendo o desenvolvimento de uma deformidade dentofacial classe III que geralmente é corrigida pela CO^(1-4)^. A deformidade dentofacial proporciona ajustes articulatórios na fala para compensar as alterações morfológicas, causando prejuízos na fala como distorção na produção dos sons e ceceios^(5-8)^. O retroposicionamento e a deficiência de crescimento do arco superior em relação ao arco mandibular na deformidade dentofacial classe III, pode afetar o posicionamento da língua durante a produção dos fones alveolares devido à protrusão lingual, mordida cruzada, atresia maxilar e dentes desalinhados^(5)^.

Nesse estudo observou-se interposição de língua, distorções, ceceio anterior e ceceio lateral; além dos distúrbios obrigatórios e articulações compensatórias presentes nos pacientes com fissura labiopalatina avaliados. Os achados mais relevantes e que tiveram melhora significativa na produção do fone após a CO foram a presença de interposição de língua e ceceio anterior. A literatura mostra que os sons fricativos são os que apresentam mais alterações nos indivíduos com deformidade dentofacial^(8,29)^, o que está de acordo com esse estudo, uma vez que todos os fones fricativos apresentaram ceceio anterior, sendo eles os alveolares [s] e [z] e os alveolopalatais [ʃ] e [Ʒ]; sendo o fone [Ʒ] sem diferença significativa de melhora após a CO. Ainda nos fricativos, houve distorção nos labiodentais [f] e [v], como observado em outro estudo^(7)^. Observou-se também melhora após a CO na interposição de língua nos fones plosivos alveolares [t], [d], no nasal alveolar [n], no lateral alveolar [l] e lateral palatal [ʎ]. Acredita-se que a mudança oclusal após a CO, favoreceu a melhora da produção dos fones.

Sabe-se que pacientes com deformidades dentofaciais apresentam alterações oclusais e musculares que provocam prejuízos no desempenho das funções orofaciais^(5,6)^. Pacientes com deformidade dentofacial Classe III apresentam maior prevalência de erros articulatórios e distorções em consoantes quando comparados a indivíduos sem alterações; sendo que a gravidade da má oclusão pode apresentar correlação com o grau de distorção da fala^(11)^. Um dos objetivos desse trabalho foi verificar se as alterações fonéticas apresentadas pelo paciente com fissura labiopalatina antes da CO tiveram melhora após a cirurgia. De acordo com esta análise, observou-se melhora significativa, o que mostra que a melhora oclusal proporcionada pela CO interferiu positivamente na fala desses pacientes que apresentaram interposição de língua, distorções e o ceceio anterior. Porém, apesar da melhora ter apresentado diferença estatisticamente significativa, a porcentagem máxima de melhora foi de 39% na interposição de língua no fone [d], ou seja, nenhum fone melhorou 100% apenas com o ajuste oclusal proporcionado pela CO, evidenciando que o trabalho fonoaudiológico é importante após a CO.

O estudo de Alaluusua et al.^(6)^, avaliou o efeito do avanço maxilar nas consoantes alveolares finlandesas [s], [l] e [ɾ] em 59 pacientes com fissuras labiopalatinas submetidos à CO. Houve melhora significativa nos fones [s] (34% dos pacientes tiveram alteração na produção do fone no pré-operatório e 20% no pós), no [l] (34% dos pacientes tiveram alteração no pré operatório e 19% no pós), enquanto o [ɾ] não apresentou diferença após o avanço maxilar. Os autores concluíram que ao planejar a CO em pacientes com fissura com retrusão maxilar e alterações fonéticas, o avanço da maxila pode ser um meio para melhorar a articulação dos fones [s] e [l] e reforçaram a necessidade de fonoterapia após a cirurgia.

Outra alteração fonética pesquisada foi o distúrbio obrigatório, que é uma consequência da disfunção velofaríngea e é caracterizado pela presença de hipernasalidade, fraca pressão intraoral e emissão de ar nasal^(25)^. Na análise estatística do presente estudo, não foi observado diferença significativa quando os distúrbios obrigatórios foram comparados isoladamente por fones, porém notou-se diferença significativa na comparação da soma total de pacientes, ou seja, após a CO, maior número de pacientes o apresentaram. Estudos mostram que após a CO de avanço de maxila, pacientes podem apresentar o aparecimento ou agravamento da hipernasalidade^(14-17)^.

Reforça-se, ainda, que um menor número de pacientes apresentou ceceio lateral após a CO, porém essa diferença não foi significativa. Estima-se que resultados diferentes podem ser encontrados em futuros estudos realizados com uma amostra maior.

Os pacientes participantes do estudo tiveram pouca presença de articulações compensatórias, fato que pode ser justificado pela participação de pacientes adultos que já passaram pelas cirurgias primárias para a correção da fissura labiopalatina e outras intervenções que podem ter auxiliado na superação ou não desenvolvimento das articulações compensatórias. Outra hipótese, é que esses pacientes já apresentavam o fechamento velofaríngeo adequado antes da CO, o que pode ter favorecido o não desenvolvimento das articulações compensatórias. Sabe-se que, aproximadamente, 30% das crianças com fissura labiopalatina apresentam disfunção velofaríngea que podem levar à hipernasalidade, fraca pressão intraoral, emissão de ar nasal e articulações compensatórias^(30)^.

Em relação aos aspectos oclusais, não se obteve diferença significativa comparando as médias de trespasse vertical pré e pós cirúrgico, o que mostra que a maioria dos pacientes apresentaram os valores médios de trespasse vertical dentro da normalidade tanto antes como após a CO (média antes: 1,37; média após: 1,89). Porém, é importante ressaltar, que os pacientes que apresentaram alterações oclusais relacionadas às medidas de trespasse vertical, tais como mordida aberta (8 pacientes, 17%) e sobremordida (10 pacientes, 21%), apresentaram melhora significativa da oclusão após a CO, ou seja, apenas 2 pacientes (4%) continuaram apresentando mordida aberta e sobremordida após a CO.

Fato semelhante ocorreu na comparação pré e pós cirurgia do trespasse horizontal, em que notou-se que as alterações oclusais de mordida cruzada anterior (42 pacientes, 88%) foram corrigidas após a CO, apresentando melhora significativa, sendo que após a cirurgia, apenas 1 paciente (2%) apresentou mordida cruzada anterior. Em relação ao trespasse horizontal aumentado (sobressaliência), inicialmente, 3 pacientes apresentavam essa alteração e após a cirurgia 19 pacientes (40%) apresentaram. Esse fato está relacionado à correção cirúrgica da deformidade dentofacial classe III. O reposicionamento do segmento da maxila e/ ou mandíbula, pode ter ocasionado um aumento do trespasse horizontal com medidas acima de 3mm que são consideradas alteradas. Além disso, os pacientes melhoraram a média de trespasse horizontal comparado à média pré cirúrgica (média antes da cirurgia: -4,54 e após 2,67 – dentro dos valores de normalidade). Os pacientes que participaram desse estudo e que apresentaram alteração oclusal de mordida em topo não tiveram mudanças oclusais significativas quando comparados pré e pós CO.

Em uma revisão sistemática em pacientes sem fissura labiopalatina submetidos à CO, verificou-se que houve diminuição do trespasse horizontal aumentado (sobressaliência) em pacientes com deformidade dentofacial classe III esquelética após a CO e em seguimento de longo prazo. A mordida aberta (*overbite*) aumentou em pacientes com deformidade dentofacial classe II, enquanto os pacientes classe III apresentaram resultados variáveis^(31)^.

Ainda no presente estudo, observou-se que houve influência das alterações oclusais verticais nos fones [ʎ] e [f] após a correção cirúrgica. Nas alterações oclusais horizontais, o ajuste oclusal proporcionado pela cirurgia melhorou a produção dos fones [t], [ʎ], [f], [v], [z] e [ʃ] . Na literatura, não foram encontrados trabalhos que enfatizassem exclusivamente esses aspectos, porém, sabe-se que as discrepâncias de trespasse vertical e horizontal podem influenciar negativamente a fala com distorções^(11)^. Hipotetiza-se que, apesar do tamanho da amostra, foram verificadas diferenças significativas principalmente relacionadas à correção cirúrgica do trespasse horizontal, o que merece ser explorado em futuros trabalhos com um número maior de indivíduos, a fim de verificar se mais fones apresentariam diferenças significativas nesses aspectos.

Neste estudo, foram utilizados os exames objetivos de nasometria e a avaliação aerodinâmica da fala. Na comparação da avaliação nasométrica da fala pré e pós CO, observou-se diferença estatisticamente significativa (p≤0,05) tanto no texto com predominância nasal, como oral, conforme já visto em outros trabalhos^(3,14)^, os quais mostraram também aumento da hipernasalidade no texto oral após o avanço de maxila.

Para a avaliação aerodinâmica da fala, utilizou-se a produção do vocábulo “rampa” nos períodos pré e pós-operatórios; e não houve diferença estatisticamente significativa (p≤0,05). No estudo de Alaluusua et al.^(3)^ o risco do paciente com fissura palatina desenvolver uma inadequação velofaríngea após o avanço de maxila é maior em pacientes que apresentam sintomas de inadequação velofaríngea antes da CO de avanço maxilar. Nesse estudo, 73% dos pacientes apresentavam fechamento velofaríngeo adequado antes da cirurgia e após 63% dos pacientes, não resultando em uma diferença estatisticamente significativa.

Pesquisadores desenvolveram um estudo^(14)^ com o objetivo de avaliar o impacto da CO na nasalância e na avaliação aerodinâmica de fala de pacientes com fissura labiopalatina. Os pacientes com fissura operada foram avaliados em 3 momentos: antes da cirurgia, 45 dias após e 9 meses após, sendo observado aumento significativo na nasalância média após 45 dias e 9 meses, comparado com o pré-operatório de sentenças orais e frases nasais. Na avaliação aerodinâmica da fala, foi observado um aumento significativo na área de fechamento velofaríngeo mínimo após 45 dias; e houve um aumento significativo na área transversal média após 9 meses da CO, com 73% dos pacientes não apresentando mais áreas subnormais vistas no pré-operatório. Concluiu-se que, a longo prazo, a CO modifica a nasalância de alguns pacientes com fissura, a qual pode ser justificada pelo aumento no tamanho do nariz, o que pode também melhorar a permeabilidade nasal para a respiração.

Em outro estudo^(19)^, foi realizada a análise dos escores de nasalância de indivíduos com fissura palatina submetidos à CO de avanço de maxila. Os autores concluíram que o aparecimento da hipernasalidade após a CO, em indivíduos com fissura de palato envolvendo ou não o lábio, ocorreu em proporções similares independentemente do tipo de fissura.

Em estudo recente^(13)^, do mesmo grupo de pesquisadores, foi realizado um levantamento nos últimos 20 anos em 535 pacientes de um centro de referência no tratamento de fissura labipalatina. O objetivo foi verificar o efeito da CO nas dimensões nasais e correlacioná-las com sintomas respiratórios. Foi observado que após a CO, houve um aumento significativo na área transversal nasal avaliada pela rinomanometria anterior e posterior, porém não houve aumento significativo na área transversal nasofaríngea. Em relação aos sintomas respiratórios após a CO, 26,3% apresentaram melhora da obstrução nasal, 28,5% na respiração oronasal, 18,5% no ronco e 5,2% na obstrução respiratória durante o sono.

Em uma revisão sistemática^(23)^, os autores tiveram o objetivo de avaliar a fala e as mudanças da função velofaríngea em pacientes com fissura palatina após cirurgia de avanço maxilar; e observou-se que o efeito do avanço maxilar na fala e no fechamento velofaríngeo ainda são controversos merecendo mais estudos delineados.

Nesta pesquisa não foi observada influência entre a oclusão (trespasse vertical e horizontal) e avaliação nasométrica; e entre os aspectos oclusais e a avaliação aerodinâmica da fala na produção do vocábulo “rampa”. Acredita-se que as mudanças oclusais proporcionadas pela CO não influenciaram os exames objetivos de nasometria e avaliação aerodinâmica da fala comprovando a hipótese inicial, de que a oclusão influencia os aspectos fonéticos (ceceios e distorções) que dependem do posicionamento dos dentes, mas não a nasalidade e fechamento velofaríngeo que dependem das estruturas musculares situadas em região de nasofaringe.

Esse estudo é inédito e explorou as modificações dos aspectos fonéticos, nasométricos e aerodinâmicos da fala em indivíduos com fissura labiopalatina e a influência da oclusão nesses aspectos, antes e após a CO de avanço de maxila. Trata-se de uma análise completa da produção de 19 sons para a análise das alterações e suas modificações proporcionadas pelo avanço maxilar. Além disso, foram analisados os exames objetivos de nasometria e a avaliação aerodinâmica da fala nos casos de pacientes submetidos ao avanço maxilar relacionando-os aos aspectos oclusais, o que foi um diferencial estudado.

Assim, fica estabelecido o perfil do paciente no pré e pós-operatório de CO para avanço da maxila, o que favorecerá o tratamento desse paciente, minimizando e prevenindo as alterações em cada etapa. Acredita-se que esses achados venham a preencher uma lacuna da literatura, porém futuros estudos com uma amostra de pacientes maior em cada tipo de fissura labiopalatina podem ser realizados a fim de verificar possíveis diferenças estatísticas que não foram encontradas neste estudo.

## CONCLUSÃO

Houve modificações dos aspectos fonéticos após a CO. As alterações fonéticas que apresentaram melhora significativa após a CO foram a interposição de língua nos fones [d], [t], [l], [n], [ʎ]; a distorção nos fones [f] e [v] e o ceceio anterior nos fones [s], [z] e [ʃ]. Em relação aos aspectos oclusais, o trespasse horizontal atingiu os valores de normalidade após a CO e houve melhora oclusal da mordida cruzada anterior, mordida aberta e sobremordida.

Na comparação da avaliação nasométrica, observou-se predominância de ressonância equilibrada após a CO para o texto nasal e houve aumento da hipernasalidade no texto oral após a cirurgia. Não houve modificação no fechamento velofaríngeo na produção do vocábulo “rampa”, sendo que este permaneceu adequado tanto no período pré como pós-operatório.

A oclusão não influenciou a nasalância e o fechamento velofaríngeo nem antes e nem após a CO de avanço de maxila.

É importante ressaltar que apesar da melhora na produção dos fones após o ajuste oclusal pela correção cirúrgica, em nenhum fone se obteve 100% de adequação, reforçando a importância da fonoterapia após a CO.
